# Association between Allergic Rhinitis and Asthma Control in Peruvian School Children: A Cross-Sectional Study

**DOI:** 10.1155/2013/861213

**Published:** 2013-07-25

**Authors:** Justo Padilla, Mónica Uceda, Otto Ziegler, Felipe Lindo, Eder Herrera-Pérez, Luis Huicho

**Affiliations:** ^1^Instituto Nacional de Salud del Niño and Universidad San Martin de Porres, Lima, Peru; ^2^Universidad San Martin de Porres, Lima, Peru; ^3^Instituto Nacional de Salud del Niño and Universidad Nacional Mayor de San Marcos, Lima, Peru; ^4^Instituto Nacional de Salud del Niño, Universidad Peruana Cayetano Heredia and Universidad Nacional Mayor de San Marcos, Lima, Peru

## Abstract

*Background*. Asthma and allergic rhinitis are highly prevalent conditions that cause major illness worldwide. This study aimed to assess the association between allergic rhinitis and asthma control in Peruvian school children. *Methods*. A cross-sectional study was conducted among 256 children with asthma recruited in 5 schools from Lima and Callao cities. The outcome was asthma control assessed by the asthma control test. A score test for trend of odds was used to evaluate the association between allergic rhinitis severity and the prevalence of inadequate asthma control. A generalized linear regression model was used to estimate the adjusted prevalence ratios of inadequate asthma control. *Results*. Allergic rhinitis was present in 66.4% of the population with asthma. The trend analysis showed a positive association between allergic rhinitis and the probability of inadequate asthma control (*P* < 0.001). It was associated with an increased prevalence of inadequate asthma control, with adjusted prevalence ratios of 1.53 (95% confidence interval: 1.19−1.98). *Conclusion*. This study indicates that allergic rhinitis is associated with an inadequate level of asthma control, giving support to the recommendation of evaluating rhinitis to improve asthma control in children.

## 1. Introduction

Asthma is the most frequent chronic respiratory disease in childhood in many regions, including developing countries, and it is thus a serious public health problem with high morbidity and economic burden [1]. The International Study for Asthma and Allergy in Childhood in its third phase showed a trend of overall prevalence increase in several countries in the last decades. However, Peru registered a decline from 26 to 19.6% from the first to the third phases [2]. A previous study conducted in a poor urban setting of Lima reported a prevalence of 20.7% [3].

On the other hand, allergic rhinitis (AR) has emerged in the last decade as a major public health problem among the allergic diseases, due to its high prevalence, its negative impact on quality of life, and its frequently associated comorbidity [4].

Asthma has several common characteristics with AR [5]. In addition, asthma is more common among AR patients [6], and it is a recognized risk factor for development of asthma in adults and in children [4, 7, 8]. Therefore, it has been suggested that both conditions would be different manifestations of a common pathogenic phenomenon of airways, representing a continuum of the same illness, although the mechanisms involved are not fully known [9].

Recent clinical guidelines highlight asthma control as the main therapeutic goal. In 2006, the Global Initiative for Asthma included the disease control level as part of the classification system for asthmatic patients [10]. However, despite the implementation of multiple diagnostic and therapeutic strategies, an adequate asthma control is still an unfulfilled goal in many patients [11]. Within this context, AR has been recognized as a cause of reduced response to asthma treatment [12] and as a prognostic factor for asthma control in adults [13]. 

The studies assessing the impact of AR severity on the level of asthma control in children are scarce, and their results are not consistent [14–17]. Even fewer have reported a standardized assessment of asthma control by using either the asthma control test (ACT) [16] or the asthma control questionnaire [14, 17].

In Peru, there are few studies evaluating the relationship between AR and asthma control [18, 19]. One showed that 54% of patients aged 2 to 14 years attending emergency services due to asthmatic exacerbations had AR, suggesting a relationship between presence of AR and severity of asthma [19]. It is unknown whether this pattern occurs in community settings such as schools, where a greater prevalence of less severe asthma would be expected.

The purpose of this study was to evaluate the association between AR and the asthma control in Peruvian school children.

## 2. Methods

### 2.1. Study Design, Setting, and Participants

**Figure 1:**
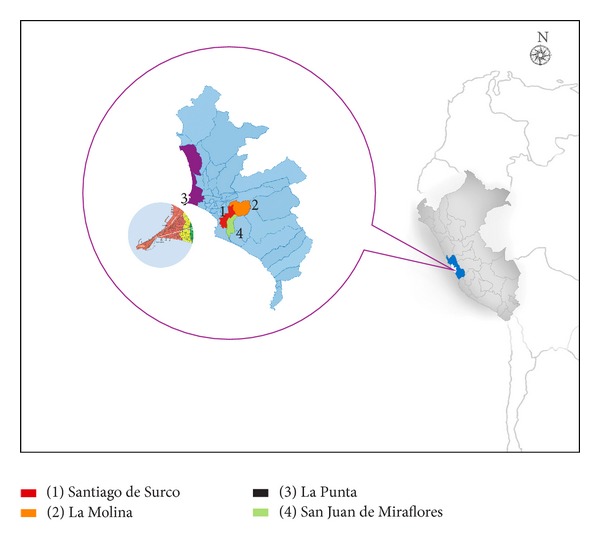
Districts of Lima and Callao where study schools were located.

We performed a cross-sectional study in asthmatic school children without relevant medical history other than asthma, from 4 schools of Lima and Callao (Figure 1), and aged 6 to 14 years. The study period was from April to May 2007. The study schools were located in four urban districts, namely, Santiago de Surco, La Molina, La Punta, and San Juan de Miraflores (Figure 1).

### 2.2. Measurements and Study Procedures

The diagnosis of asthma was made by a trained member of the research team, using the diagnostic questionnaire for the International Primary Care Respiratory Group Guidelines (IPCRG) among children with previous symptoms and/or diagnosis of asthma. We excluded children with birth defects and chronic diseases (cystic fibrosis, tuberculosis, deficiency of alpha 1 antitrypsin, and heart diseases), those with any condition causing recurrent bronchial obstruction (chronic rhino-sinusitis, gastroesophageal reflux, recurrent lower respiratory tract infections, bronchopulmonary dysplasia, foreign body aspiration syndrome, and primary ciliary dyskinesia), and those who had been enrolled currently in another research study. Comorbidities were ruled out on the basis of clinical history and examination. 

Asthma and AR were diagnosed according to the IPCRG diagnostic questionnaire [20]. The AR severity was diagnosed according to the allergic rhinitis and the impact on asthma (ARIA) tool [21].

We categorized the different levels of asthma control according to the ACT tool, depending on different score cut-off levels by age. Children younger than 12 years old with a score of 19 or less were classified as having inadequately controlled asthma [22]. Children of 12 years and older were classified as follows: completely controlled asthma for a score of 25, partially controlled asthma for a score from 21 to 24, and not controlled asthma for a score lower than 21 [23].

Based on the results of the IPCRG questionnaire for asthma diagnosis, we excluded children without asthma. Then, we administered the IPCRG questionnaire for the diagnosis of AR only to children with asthma diagnosis. Accordingly, we obtained two groups: asthmatic patients with and without AR. A third questionnaire was applied only to children with asthma and AR (ARIA questionnaire for severity assessment of AR). Finally, a fourth questionnaire (ACT) was applied for assessing the control level of asthma. 

Children under 12 years old were interviewed along with their parents, while children older than 12 years old were interviewed alone.

The variable level of asthma control was dichotomized for patients with 12 years and older in two categories, that is, inadequate asthma control (partially and not controlled asthma) and adequate asthma control (completely controlled asthma). For children younger than 12 the categories remained the same (inadequately and adequately controlled asthma). 

The socioeconomic status was defined according to school expenses quintiles as richest; richer; middle; poorer/poorest, in agreement with the classification used by the National Institute of Statistics and Computing [24].

### 2.3. Sample Design

For sample size calculation we used the following parameters: asthma prevalence of 20% [3], 95% of confidence level, and 5% of accuracy level. A sample size of 246 was expanded to 270 allowing for 10% of potential data missing. We distributed the total sample size proportionally to size of socioeconomic strata of the population from Lima and Callao cities.

### 2.4. Data Analysis

Demographic and clinical data were compared by age group using the chi-square test. Prevalence of inadequate asthma control was computed and compared based on demographic and clinical characteristics through the Pearson chi-square tests. Similar to the study by De Magalhães et al., we used a chi-square trend test (score test for trend of odds) for evaluating the effect of AR severity on the prevalence of inadequate asthma control [15].

We constructed a multivariate binomial logistic generalized linear model by using inadequate controlled asthma as the dependent variable and AR coexistence, sex, age group, and socioeconomic status as independent variables. We used Bayesian and Akaike's information criteria to identify the statistical model that could best explain an inadequate asthma control, by considering the statistically significant covariables obtained through univariate analysis.

All analyses were performed using Stata software, version 12.0 (StataCorp LP, College Station, TX, USA). Values of *P* < 0.05 were considered statistically significant.

### 2.5. Ethics

Institutional permission was requested from the principals of participating schools. All parents of eligible children and children older than 7 years old provided informed verbal consent. This study was approved by the Ethics Committee of Instituto Nacional de Salud del Niño, Lima, Peru.

## 3. Results

We enrolled 256 asthmatic children. Their mean age was 9.7 years old (boys 9.9 years old and girls 9.6 years old). The age range was from 7 to 17 years old. All patients were from mestizo ethnic background.  Table 1 shows basic sociodemographic and clinical characteristics by age group.

Neither gender nor socioeconomic status was significantly associated with asthma control, while AR was significantly associated with a lack of adequate asthma control (Table 2).

**Figure 2:**
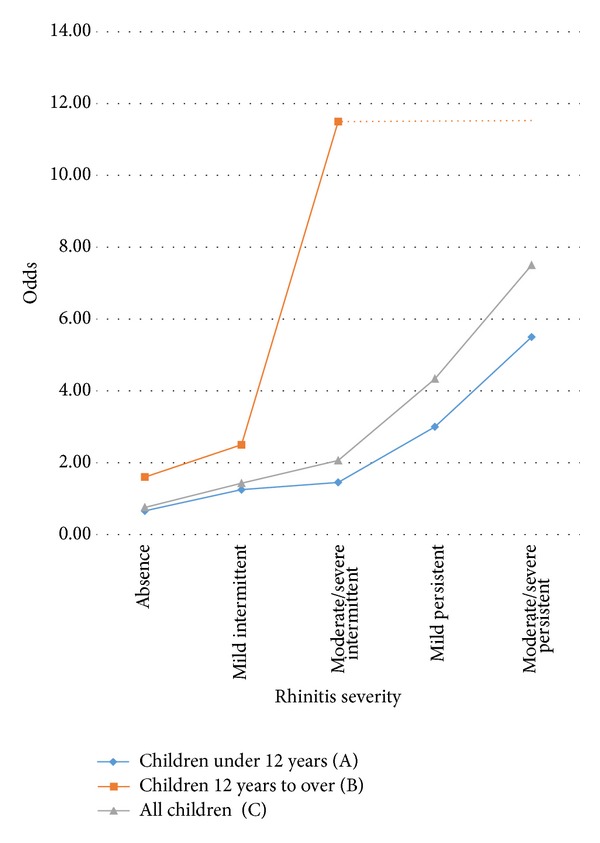
Odds tendency to inadequate control of asthma in 256 asthmatic children, by age group. *P*  value for odds homogeneity test: A = 0.007, B = 0.097, and C ≤ 0.001; *P* value to the score test with tendency of odds: A ≤ 0.001, B = 0.008, and C ≤ 0.001. The dotted line describes a predicted trend (see text).

The trend analysis showed a positive association between AR severity and the probability of inadequate asthma control (expressed as odds), particularly among children of 12 years and older (Figure 2). The trend is shown with dotted lines for subjects 12 years and older with moderate/severe, mild persistent, or moderate/severe AR, because there were no subjects with adequate asthma control. The best model for explaining an inadequate asthma control, which showed the lowest criteria values, was the one that included age and AR (Table 3). It explained 24.13% of the inadequate asthma control variance.

The prevalence of inadequate asthma control adjusted for age was 53% higher in asthmatic children with concomitant AR than that in asthmatic children without concomitant AR (prevalence ratio 1.53, 95% CI 1.19–1.98; *P* = 0.001).

According to our statistical power estimation, we needed 170 children in the group with inadequate control of asthma and 86 in those with adequate control. This allowed a 98% power to detect a prevalence ratio of 1.39 for an inadequate control of asthma [15].

## 4. Discussion

We found that AR is significantly associated with inadequate asthma control, irrespective of age. Neither gender nor socioeconomic status influenced the level of asthma control. Our results are in line with previously reported studies.

Several studies on outpatients have shown that AR is the allergic disease mostly associated with asthma [25]. It results in a lower resolution of airway obstruction [12], increased frequency of asthma exacerbations and/or severity of asthma disease [26], higher probability of severe asthma [27] and uncontrolled asthma [12], increased school absence [28], increased visits to emergency rooms [12, 27, 29], more frequent hospitalization [29, 30], lower quality of life [17], higher healthcare cost [25, 29], and higher demand for health services [29].

Treatment of AR may contribute to the prevention or improvement of coexisting asthma [9, 12]. Lack of AR control in asthmatic patients can lead to an increase of rescue or preventive asthma medication [31]. This might explain, at least in part, that only 2.4% of Latin American asthmatic patients have a totally controlled asthma [32].

Previous studies in Peru have reported AR coexistence in asthmatic children between 2 and 14 years old, suggesting that it would be an aggravating factor in asthmatic disease and vice versa [19]. Although the researchers found a significant association of AR with frequency and severity of asthma exacerbations, the asthma control level was not formally assessed [18].

The deleterious effect of AR on the level of asthma control seemed to increase with age in our study. While it is possible that this is showing a cumulative effect of AR on the asthmatic condition over time, it could also be a consequence of the cutoffs we used for categorizing children in two age groups. Previous studies did not find a significant association between age and asthma control [14–17, 33]. However, they did not assess reliably this association through the use of an appropriate predictive model like the one we used.

Prevalence of AR varies from 80% to 90% in asthmatic patients [34, 35]. In Latin America, the prevalence of isolated AR (without asthma coexistence) is up to 20% [36], and in asthmatic patients it ranges from 28% [36] to 95% [37]. About 13% to 38% of AR patients are asthmatic, compared with 5% to 15% observed in the general population [36]. In our study more than 66% of asthmatic children had AR, a prevalence similar to that reported in Cuba [38].

In our study setting, AR prevalence varies between 47% [18] and 54% [39] in patients attending pediatric emergency facilities due to asthmatic exacerbations. In our school-based study we found a higher proportion of AR-asthma coexistence. Although these results could show an increased proportion of AR in asthmatic patients, it could also reflect age differences between the study populations and different diagnostic criteria used.

We acknowledge some limitations in our study. First, the sample design used is not representative, and therefore we need caution before generalizing our results. Second, the results may not be applicable to preschool children and children from rural settings. Third, differences due to genetic and environmental factors were not considered. Fourth, we did not evaluate the treatment received or the adherence to management in our patients. However, one characteristic that we notice regularly in our practice setting is that the great majority of asthmatic patients do not receive inhaled corticosteroids. On the contrary, virtually all receive inhaled or nebulized beta-agonists and systemic corticosteroids (prednisone or prednisolone) when they present acute exacerbations. These two facts likely reduced the effects of treatment heterogeneity on asthma control in our study subjects. Finally, the cross-sectional design precluded assessing any seasonal trend.

Previous studies performed logistic regression analysis to assess the association between AR and asthma control [14–17]. However, prevalence odds ratios can substantially overestimate the prevalence ratio when working with frequent outcomes [40]. In turn, the prevalence ratio is conservative, consistent, and more appropriate to interpret, and it should be used in preference to the prevalence odds ratio [41]. Thus our generalized linear model is an appropriate alternative for performing the multivariate estimation of the prevalence ratio.

This is the first paper reporting the possible effect of AR on asthma control in a Peruvian school community from Lima and Callao. This is particularly relevant, considering that AR is underdiagnosed at primary level health facilities in Peru [18, 19] and other Latin American countries [38, 42–45]. Our proposed explanatory model is powerful enough for reliably explaining factors related to an inadequate control of asthma.

## 5. Conclusions

Our study confirms that AR coexistence in asthmatic patients is associated with inadequate asthma control. This may have implications on the management of these diseases. We recommend the regular seeking of AR coexistence when evaluating asthmatic children, particularly among those with difficult-to-treat asthma. Further investigations are required to determine whether appropriate treatment of AR can efficiently reduce asthma morbidity.

## Figures and Tables

**Table 1:** Sociodemographic and clinical characteristics of 256 asthmatic children.

Characteristics	Children under 12 years (*n* = 201)	Children 12 years or older (*n* = 55)	*P* value*	All children (*n* = 256)
	*n* (%)	*n* (%)		*n* (%)
Female	93 (46.27%)	20 (36.36%)	0.19	113 (44.14%)
Socioeconomic status				
Richest	12 (5.97%)	14 (25.45%)	<0.01	26 (10.16%)
Rich	31 (15.42%)	12 (21.82%)	0.60	43 (16.80%)
Middle	75 (37.31%)	3 (5.45%)	<0.01	78 (30.47%)
Poorer/poorest	83 (41.29%)	26 (47.27%)	Ref.	109 (42.58%)
Presence of allergic rhinitis	128 (63.68%)	42 (76.36%)	0.08	170 (66.41%)
Inadequate asthma control	109 (54.23%)	35 (63.64%)	<0.01	155 (60.55%)

*Chi-square test; Ref: reference level for analysis.

**Table 2:** Factors associated with inadequate asthma control in 256 asthmatic children.

Factor	Inadequate asthma control		*P* value*
	Yes (*n* = 170)	No (*n* = 86)	
	*n* (%)	*n* (%)	
Female	70 (44.16%)	43 (42.57%)	0.68
Age: 12 years or older	46 (29.68%)	9 (8.91%)	<0.01
Socioeconomic status			0.29
Richest	16 (10.32%)	10 (9.90%)	
Rich	24 (15.48%)	19 (18.81%)	
Middle	42 (27.10%)	36 (35.64%)	
Poorer/poorest	73 (47.10%)	36 (35.64%)	
Presence of allergic rhinitis	118 (76.13%)	52 (51.49%)	<0.01

*Chi-square test.

**Table 3:** Models to explain an inadequate asthma control.

Statistical models	LL	DF	Criterion used	
			AIC	BIC
N0: null	−171.71	1	345.41	348.96
N1: rhinitis	−163.45	2	330.90	337.99
N1: age	−162.74	2	329.47	336.56
N2: rhinitis and age	−154.79	3	315.58	326.22

Null: model that includes only the outcome variable (inadequate controlled asthma); N1: model with one covariable; N2: model with two covariables; LL: log likelihood; DF: degrees of freedom; AIC: the Akaike Information criterion; BIC: the Bayes information criterion.
